# Draft genome sequence and tissue expression panel of Pacific saury (*Cololabis saira*)

**DOI:** 10.1093/dnares/dsae010

**Published:** 2024-04-03

**Authors:** Yoji Nakamura, Motoshige Yasuike, Taiki Fuji, Satoshi Suyama, Miyuki Mekuchi

**Affiliations:** Bioinformatics and Biosciences Division, Fisheries Stock Assessment Center, Fisheries Resources Institute, Japan Fisheries Research and Education Agency, 2-12-4 Fuku-ura, Kanazawa, Yokohama, Kanagawa 236-8648, Japan; Bioinformatics and Biosciences Division, Fisheries Stock Assessment Center, Fisheries Resources Institute, Japan Fisheries Research and Education Agency, 2-12-4 Fuku-ura, Kanazawa, Yokohama, Kanagawa 236-8648, Japan; Highly Migratory Resources Division, Fisheries Stock Assessment Center, Fisheries Resources Institute, Japan Fisheries Research and Education Agency, 2-12-4 Fuku-ura, Kanazawa, Yokohama, Kanagawa 236-8648, Japan; Highly Migratory Resources Division, Fisheries Stock Assessment Center, Fisheries Resources Institute, Japan Fisheries Research and Education Agency, 25-259 Shimomekurakubo, Same, Hachinohe, Aomori 031-0841, Japan; Bioinformatics and Biosciences Division, Fisheries Stock Assessment Center, Fisheries Resources Institute, Japan Fisheries Research and Education Agency, 2-12-4 Fuku-ura, Kanazawa, Yokohama, Kanagawa 236-8648, Japan

**Keywords:** Pacific saury, transcriptome, tissue expression panel

## Abstract

Pacific saury (*Cololabis saira*) is an important fish in several countries. Notably, the catch of this fish has markedly decreased recently, which might be due to environmental changes, including feeding habitat changes. However, no clear correlation has been observed. Therefore, the physiological basis of Pacific saury in relation to possible environmental factors must be understood. We sequenced the genome of Pacific saury and extracted RNA from nine tissues (brain, eye, gill, anterior/posterior guts, kidney, liver, muscle, and ovary). In 1.09 Gb assembled genome sequences, a total of 26,775 protein-coding genes were predicted, of which 26,241 genes were similar to known genes in a public database. Transcriptome analysis revealed that 24,254 genes were expressed in at least one of the nine tissues, and 7,495 were highly expressed in specific tissues. Based on the similarity of the expression profiles to those of model organisms, the transcriptome obtained was validated to reflect the characteristics of each tissue. Thus, the present genomic and transcriptomic data serve as useful resources for molecular studies on Pacific saury. In particular, we emphasize that the gene expression data, which serve as the tissue expression panel of this species, can be employed in comparative transcriptomics on marine environmental responses.

## 1. Introduction

Fish are an essential source of animal proteins and other valuable nutrients.^1,2^ Aquatic foods provide varying levels of nutrients depending on the species. According to the Food and Agriculture Organization of the United Nations (FAO), fisheries produce more than 50% of fish consumed globally and are the main source of marine fish supplies.^3^ Pacific saury (*Cololabis saira*) is a small pelagic teleost (Scomberesocidae, Beloniformes) and an important commercial species in several countries.^4^ This species is widely distributed in the North Pacific Ocean from the subtropical to subarctic regions.^5^ Pacific saury distributed around the Japanese Archipelago mainly migrate northward from the subtropical to subarctic region in May and July and then begin returning to the south after August.^6,7^ The lifespan of Pacific saury is thought to be less than 2 years, and their puberty age is 0 years.^8,9^ Spawning occurs from September to June of the following year.^10,11^ Recently, the biomass of Pacific saury has remarkably decreased, and the alternation of distribution and migration routes has been confirmed.^12–14^ Environmental changes, including in the feeding environment, are proposed to contribute to the decline. Pacific saury feeds on boreal zooplankton, mainly *Neocalanus*, a copepod.^15^ Different phenological patterns, such as changes in blooming time, have been identified as changes to the ocean environment. However, its correlation with a poor catch has not been clearly defined. As Pacific saury is a pelagic fish, it is difficult to rear; therefore, biological and physiological studies on this fish are limited. As an initial step in collecting basic molecular information on Pacific saury, we sequenced the whole genome of this species and performed transcriptome analysis. In particular, we aimed to provide transcriptome resources for Pacific saury, which will be useful for future molecular studies examining the relationships between the reproductive process of this fish and ecological changes.

## 2. Materials and methods

### 2.1. Sampling and sequencing

Fish sampling was conducted in strict accordance with the principles and guidelines for the care and use of live fish and the guidelines for animal experimentation at Fisheries Resources Institute, Japan Fisheries Research and Education Agency. Fish (~ 70 g) were caught in a research vessel in the northwestern Pacific Ocean. Genomic DNA was extracted from a liver stored in a deep freezer at −80°C. Approximately 50 mg of the liver was digested using 6 M TNES-Urea buffer, Proteinase K (20 mg/µl), and 2 µl of RNase A (100 mg/µl) and then incubated overnight at 37°C with slow shaking. DNA was extracted from the digested samples using the standard phenol-chloroform method.^16^ The quantity and quality of the extracted DNA were measured using a Qubit® 2.0 Fluorometer (Thermo Fisher Scientific, Waltham, MA, USA) and an Agilent 2200 TapeStation system (Agilent Technologies, Palo Alto, CA, USA), respectively. Genomic DNA was sequenced using the PacBio Sequel II platform in HiFi mode (Pacific Biosciences, Inc., CA, USA) at Macrogen, Inc. (Seoul, South Korea).

For gene expression profiling, the eye, gill, anterior gut, posterior gut, liver, kidney, and muscle were sampled from the same fish for genome sequencing. The brain and ovary were sampled from another fish and immersed in an RNA stabilization solution (RNA-later, Thermo Fisher Scientific, Waltham, MA, USA). Total RNA was extracted using Direct-zol™ (Zymo Research, Irvine, CA, USA), according to the manufacturer’s protocol. The RNA quality was evaluated based on the proportion of rRNA using an Agilent 2200 TapeStation system. Complementary DNA libraries were constructed using 1 µg of total RNA and the Illumina TruSeq Stranded mRNA Library Prep Kit (Illumina, San Diego, CA, USA). Libraries were sequenced using an Illumina NextSeq 500 platform equipped with a 150 bp paired-end module.

### 2.2. Genome assembly and repeat masking

The HiFi reads of Pacific saury were assembled using Improved Phased Assembler (IPA) v.1.3.1 (https://github.com/PacificBiosciences/pbipa). Repetitive sequences were predicted and masked by RepeatMasker v4.1.3^17^ using the Pacific saury repeat library, which was constructed in advance using RepeatModeler v2.0.3.^18^ K-mer analysis for raw read sequences was conducted using JellyFish v2.3.0^19^ (*k* = 15). Completeness of the genome assembly was assessed using BUSCO v5.3.0^20^ with the Actinopterygii (ray-finned fish) ortholog set.

### 2.3. Gene prediction and annotation

The RNA-Seq reads sequenced from the nine tissues of Pacific saury were mapped to the contig sequences using HISAT2 v2.2.1^21^ after processing with Trimmomatic v0.39,^22^ and the transcripts were constructed using StringTie v2.1.6.^23^ Here, the reads were treated as 150 bp single-end because the reverse sequencing failed. The protein sequences of five model fish species, *Danio rerio*, *Gasterosteus aculatus*, *Oryzias latipes*, *Takifugu rubripes*, and *Tetraodon nigroviridis*, were downloaded from the Ensembl database release 104.^24^ Thereafter, the gene models of the Pacific saury genome were predicted using MAKER v3.01.04^25^ with the transcripts of Pacific saury and the protein sequences of model fish species. Finally, genes containing less than 50 amino acids were excluded from the analysis. The functions of the predicted genes were inferred using DIAMOND v2.0.15^26^ (*E*-value < 10^−10^) against the NCBI NR database on 16 May 2022.

### 2.4. Comparison of the gene expression profiles

The expression of each gene in the nine tissues of Pacific saury was measured as transcripts per million (TPM) using StringTie. The genes highly expressed in a specific tissue were estimated using the tissue specificity index, *τ*.^27^ After filtering out genes with TPM < 10 in all tissues as weekly expressed genes,^28^ genes with *τ* >= 0.85 were collected as tissue-specific expressed genes.^27^ To compare the gene expression profiles of Pacific saury tissues to those of model organisms, we downloaded the tissue panel data of zebrafish (*Danio rerio*) and medaka (*Oryzias latipes*) from the ArrayExpress database (project number: E-MTAB-8959; URL: https://www.ebi.ac.uk/biostudies/arrayexpress/studies/E-MTAB-8959),^29^ where the gene expression data for eight tissues, namely liver, skin, muscle, heart, gut, gill, eye, and brain, were deposited. Orthologous genes among Pacific saury, zebrafish, and medaka were estimated using OrthoFinder v.2.5.4.^30^ Here, the protein sequences of Ensembl release 88, originally used in E-MTAB-8959, were used for zebrafish and medaka. Single-copy orthologs for which TPMs were measured in all examined tissues were collected. The original project, E-MTAB-8959, also contains gene expression data for rainbow trout (*Oncorhynchus mykiss*); however, this species was not used in the present study due to the lack of original annotation data. After the TPMs were converted to log_2_(TPM+1), principal component analysis (PCA) was conducted using *R* (*prcomp*). Hierarchical clustering of tissue samples was conducted using the group average method based on cosine similarity, and the heatmap was generated using *heatmap.2* in the *gplots* library of *R*. Functional enrichment analysis of the tissue-specific expressed genes in Pacific saury was performed using the Metascape^31^ based on the Gene Ontology (GO) terms of Ensembl gene IDs of zebrafish ortholog.

## 3. Results and discussion

### 3.1. Genome assembly and assessment

**Figure 1.**
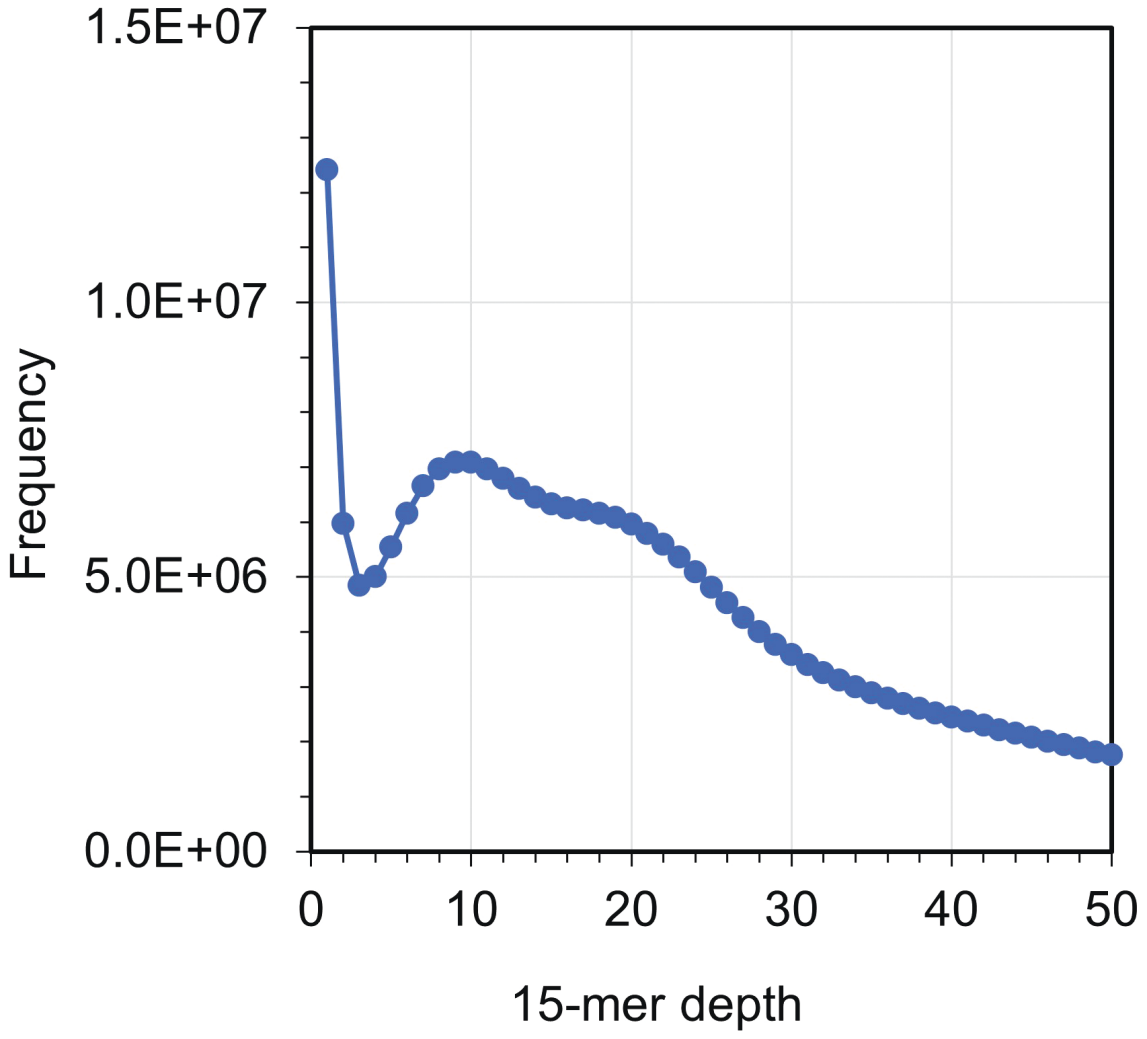
**K-mer spectrum for the whole genome shotgun reads of Pacific saury.** The x-axis denotes the 15-mer depth, while the y-axis denotes the frequency.

The *de novo* assembly of Pacific saury using 19.1 Gb HiFi reads yielded 2,910 contigs, totalling 1.09 Gb (**Table 1**). In the k-mer frequency analysis, a main peak was observed at a depth of 9; however, a hidden peak appeared around a depth of 17-19 (**Fig. 1**). As the raw read sequences may contain more errors than those from traditional sequencing, and this species might be highly heterozygous,^32^ the sequencing depth might correspond to this hidden peak. As the genome size of Pacific saury has never been reported, we compared the estimate (1.09 Gb) to the C-value-based genome size of Belonidae, a sister clade of Scomberesocidae. According to the Animal Genome Size DB,^33^ the genome size of Belonidae is approximately 1 Gb (C-values:1.00-1.20) with 2n = 48 of karyotype. As the chromosome number of Pacific saury is 2n = 42,^34^ this species is unlikely to have recently undergone genome expansion, such as polyploidization, after its divergence from the Belonidae lineage. Therefore, the DNA content of Pacific saury may be close to that of Belonidae species; that is, the genome size of Pacific saury may be close to 1 Gb, suggesting that the current assembly result is reasonable. Furthermore, in the BUSCO analysis, 94.4% of the Actinopterygii ortholog groups (single-copy: 91.3%; duplicated: 3.1%) were successfully captured from the current assembly.

**Table 1.** Assembly statistics of the Pacific saury genome

Number of contigs	2,910
Total size of contigs (bp)	1,089,720,178
Average contig length (bp)	374,474
N50 contig length (bp)	859,535
N50 contig number	311
Longest contig (bp)	6,470,421
G+C content (%)	41.5
Repeat masked length (bp)	547,205,451

### 3.2. Repetitive elements and gene annotation

**Figure 2.**
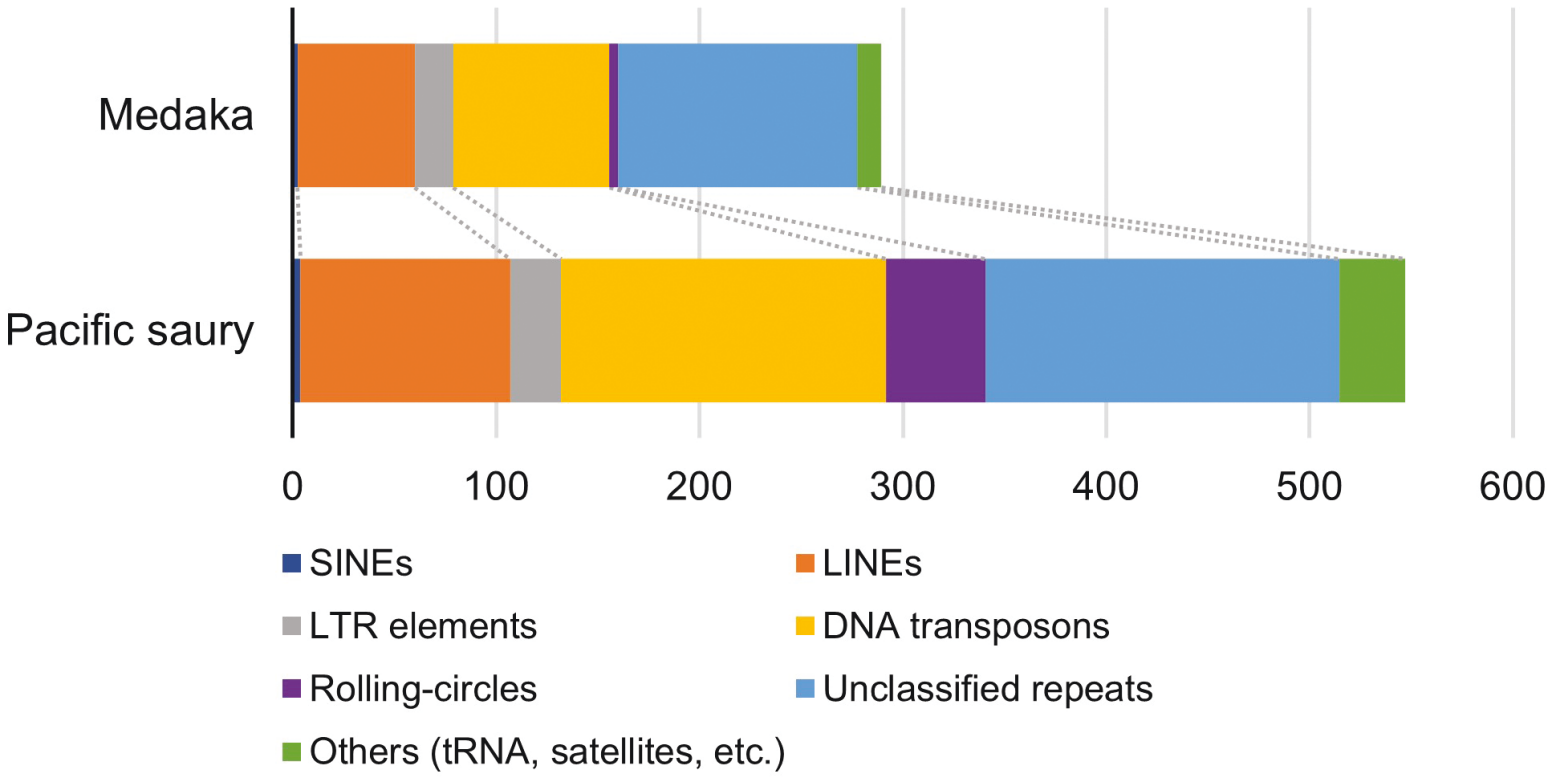
**Content of the repetitive elements predicted in the Pacific saury genome.** The abundances of the repetitive elements were compared to those in the medaka genome (Ensembl release 104), which were calculated by the same procedure as that employed for the Pacific saury genome.

In repetitive sequence analysis, approximately 50% (~0.55 Gb) of the total nucleotides were predicted to be repetitive sequences (**Table 1 and Fig. 2**). Compared with the medaka genome (~0.29 Gb out of ~0.73 Gb, Ensembl release 104), the Pacific saury genome may be relatively rich in repetitive elements. Most of the repetitive sequences were interspersed repeats (~0.51 Gb), and many of the annotated sequences were LINEs (~0.10 Gb), DNA transposons (~0.16 Gb), and unclassified repeats (~0.17 Gb). These repeats and rolling-circles (helitrons) were more abundant in the Pacific saury genome than in the medaka genome, accounting for approximately 60% (~0.23 Gb) of the difference in genome size (1.09 − 0.73 = 0.36 Gb). We note that helitrons may be a kind of DNA transposons, but here were classified into another category, namely, ‘rolling-circles’ according to the RepeatMasker output. Among the known repeats, in particular, L2 (LINE), PIF-Harbinger (DNA transposon), TcMar-Tc1 (DNA transposon), and hAT-Ac (DNA transposon) were markedly more abundant in the Pacific saury genome than in the medaka genome (**Supplementary Fig. 1**).

**Figure 3.**
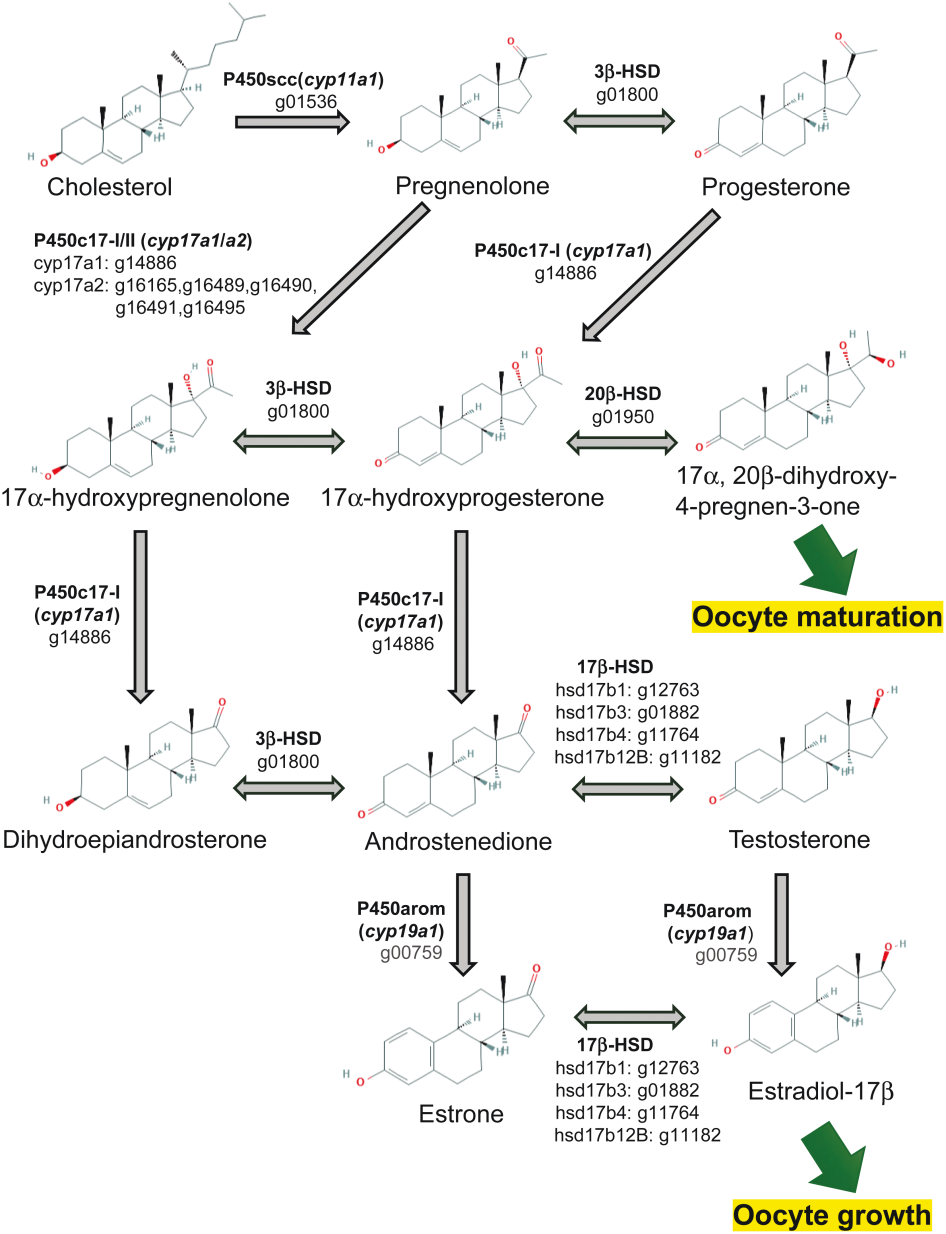
Steroidogenesis pathway of Pacific saury inferred from the gene annotation. The key enzymes in the steroidogenic pathway and their encoded genes are shown. The numbers that start with ‘g’ below the enzyme names are gene IDs of Pacific saury (Supplementary Table 1). The chemical structures are cited from PubChem.^37^ PubChem Compound Summary: CID 5997 cholesterol, CID 8955 pregnenolone, CID 5994 progesterone, CID 91451 17alpha-hydroxypregnenolone, CID 6238 hydroxyprogesterone (17alpha-hydroxyprogesterone), CID 107701 (20R)-17,20-dihydroxypregn-4-en-3-one (17alpha, 20beta-dihydroxy-4-pregnen-3-one), CID 5881 prasterone (dehydroepiandrosterone), CID 6128 androstenedione, CID 6013 testosterone, CID 5870 estrone, CID 5757 estradiol (estradiol-17beta).

In gene prediction, a total of 26,775 loci were predicted to be protein-coding regions (**Table 2**). Of these, 26,241 genes (98.0%) were matched to known protein sequences in the NCBI NR database. In the BUSCO assessment performed with the protein mode, 84.8% of the Actinopterygii proteins were identified (single-copy: 81.5%; duplicated: 3.3%). Furthermore, we estimated 12,480 ortholog groups among Pacific saury, zebrafish and medaka using OrthoFinder, and checked for reproduction-related genes in the Pacific saury genome. In this study, we identified the genes in the steroidogenesis pathway based on the previous studies examining medaka^35^ and zebrafish^36^ (**Fig. 3**). This pathway is essential for sex steroid synthesis in fish. One of the estrogens, 17β-estradiol (E2), plays a critical role in oocyte growth, and 17α,20β-dihydroxy-4-pregnen-3-one (DHP) is required for oocyte maturation.^38,39^ In this study, all of the key enzyme genes for the hormone synthetic pathway of Pacific saury were identified for the first time. Recently, a decline in the biomass of the Pacific saury has been observed.^12^ Reproduction is an influential bio-event that alternates the population. Monitoring maturation status is important for the resource conservation, and elucidation of the gonadal maturation mechanisms of the Pacific saury is an initial step in understanding the reproductive system.

**Table 2.** Predicted genes in the Pacific saury genome

Number of predicted protein-coding genes	26,775
Matched to known protein sequences	26,241 (98.0%)
Expressed in at least one tissue^a^	24,254 (90.6%)
Tissue-specific expressed^a^	7,495 (28.0%)

^a^Among nine tissues (brain, eye, gill, anterior/posterior guts, kidney, liver, muscle, and ovary).

### 3.3. Gene expression profiles

**Figure 4.**
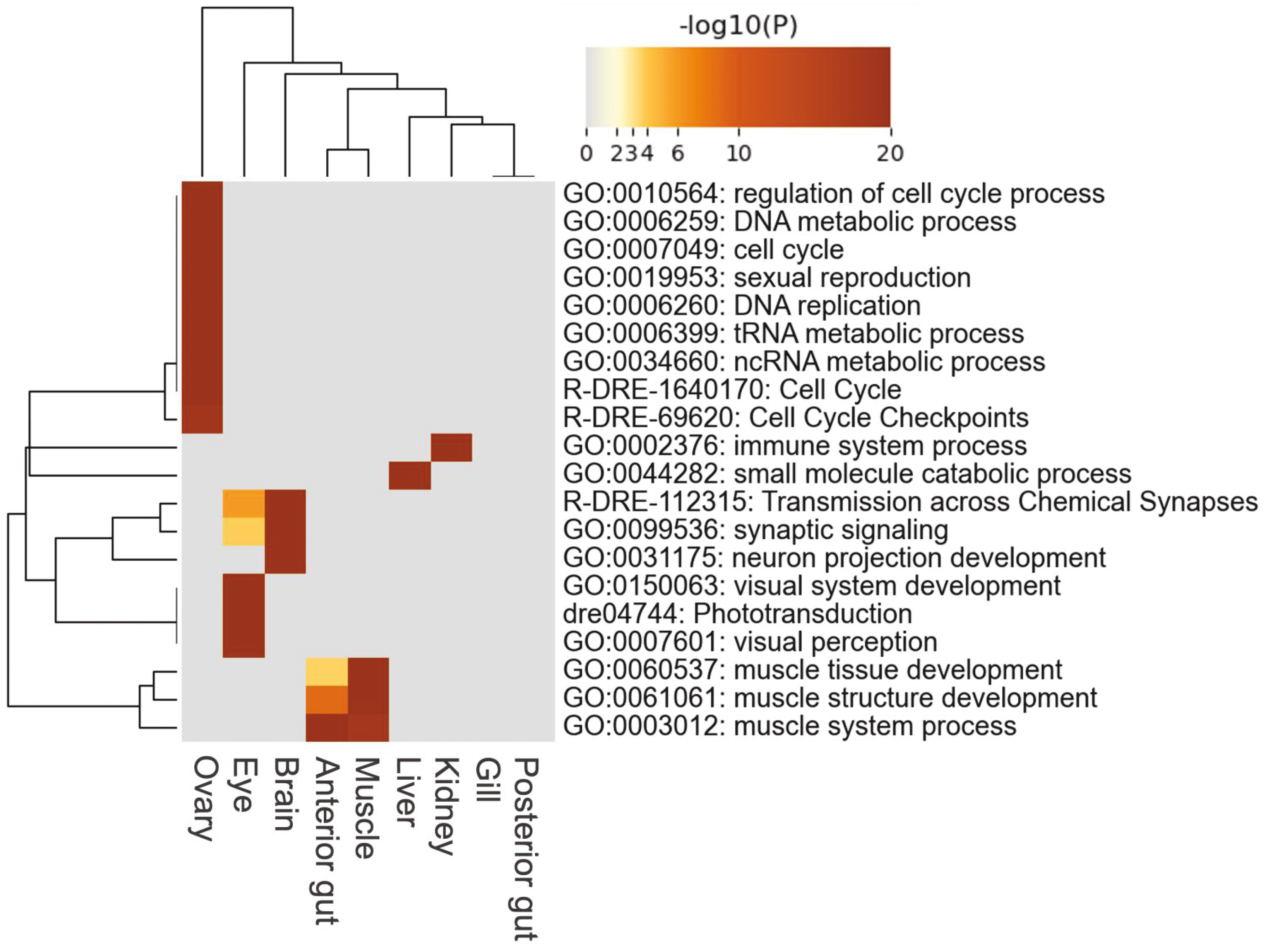
**Heatmap of the top 20 enriched GO terms across tissue-specific expressed genes of the nine tissues.** The heatmap cells are colored by their *P* values. A darker colour indicates a lower *P* value, whereas grey colour indicates the lack of enrichment for that term in the corresponding tissue.

Using RNA-Seq data, we measured the gene expression levels in nine tissues of Pacific saury, namely the brain, eye, gill, anterior gut, posterior gut, kidney, liver, muscle, and ovary. In total, 24,254 genes (90.6% of the predicted genes) were expressed (i.e. TPM > 0) in at least one tissue sample (**Table 2**). We predicted 7,495 (28.0% of the predicted genes) genes as tissue-specific expressed genes (2,477 in the brain, 769 in the eye, 383 in the gill, 306 in the anterior gut, 285 in the posterior gut, 347 in the kidney, 460 in the liver, 323 in the muscle, and 2,145 in the ovary) (**Supplementary Table 1**), which may be used to characterize each tissue in Pacific saury. We conducted a GO enrichment analysis of tissue-specific genes expressed in the Pacific saury (**Fig. 4**). The GO terms of the Pacific saury genes were assigned from those of orthologous zebrafish genes. The following GO terms associated with the functions of each tissue were significantly enriched: ‘sexual reproduction’ for the ovary, visual system-related terms for the eye, and neural transmission-related terms for the brain. Thus, the current transcriptome data accurately reflected the characteristics of Pacific saury tissues.

**Figure 5.**
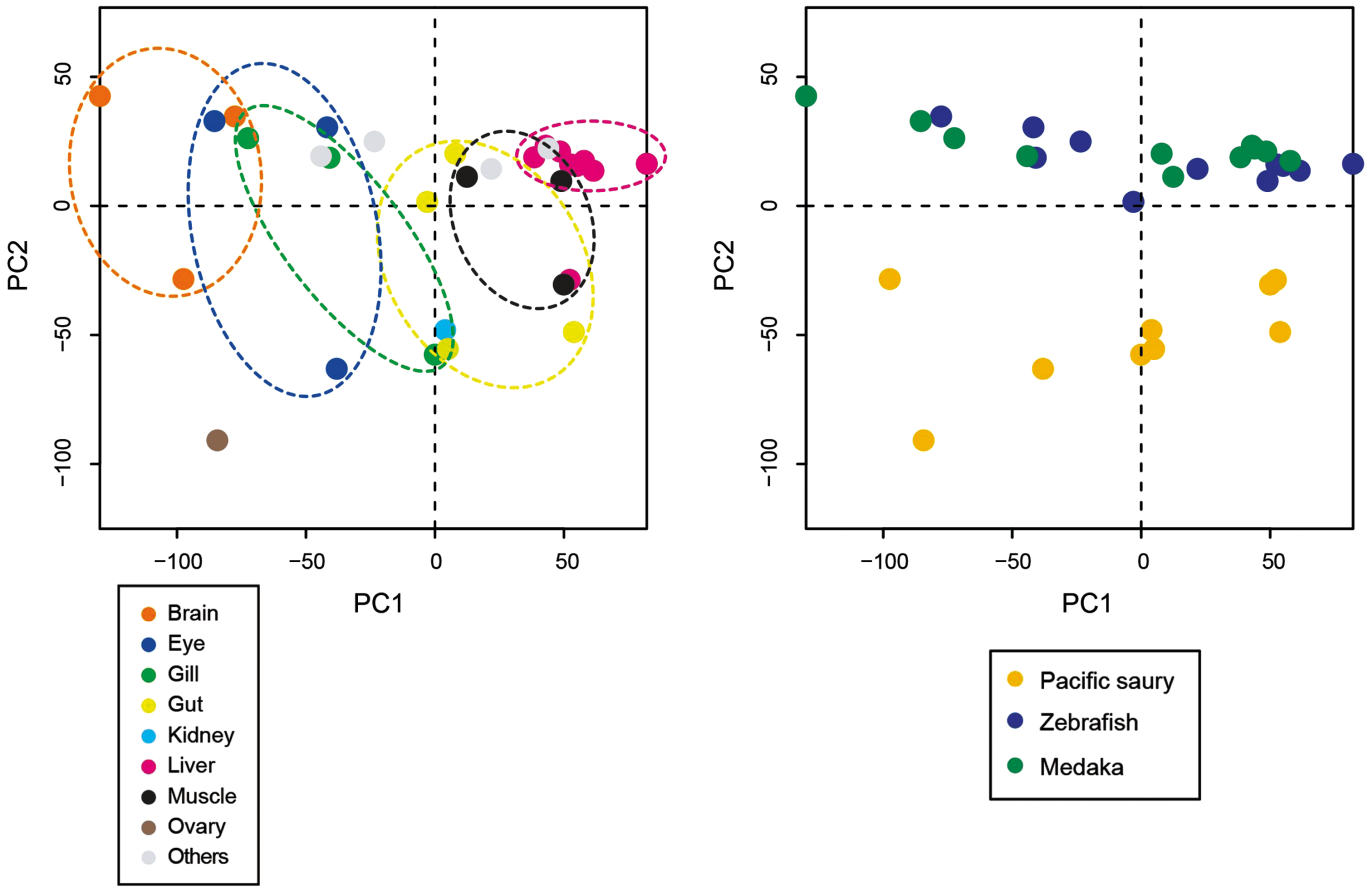
**Principal component analysis (PCA) plot of the transcriptome samples.** The expression data of 8,963 single-copy orthologs among Pacific saury, zebrafish, and medaka were used. The first two principal components (PCs) were plotted, and the same plot was colored according to tissues (A) and organisms (B), respectively. The anterior/posterior guts of Pacific saury were assigned to the ‘Gut’ category.

Furthermore, we compared the expression profiles of Pacific saury tissues with those of zebrafish and medaka. Gene expression data were available for eight tissues of zebrafish and medaka, six of which (liver, muscle, gut, gill, eye, and brain) were comparable to those of Pacific saury. As a comparative evaluation of the expression of multicopy genes is difficult to perform among species, where factors, such as dosage effects, may be considered,^40^ we focussed on single-copy genes. Of the 12,480 orthologous groups, 8,964 were single-copy genes, almost all of which (8,963) were expressed in at least one tissue of the three species. As a validation of transcriptome data, we conducted PCA using the expression data of these 8,963 genes (**Fig. 5**). PCA revealed that the first principal component may reflect the difference in expression patterns in each tissue rather than in each lineage (**Fig. 5a**), although the second principal component clearly separated Pacific saury and the model species (**Fig. 5b**). These results suggest a correspondence between the tissue samples of Pacific saury and the model species at the gene expression profile level, ensuring the quality of the transcriptome data for Pacific saury tissues.

**Figure 6.**
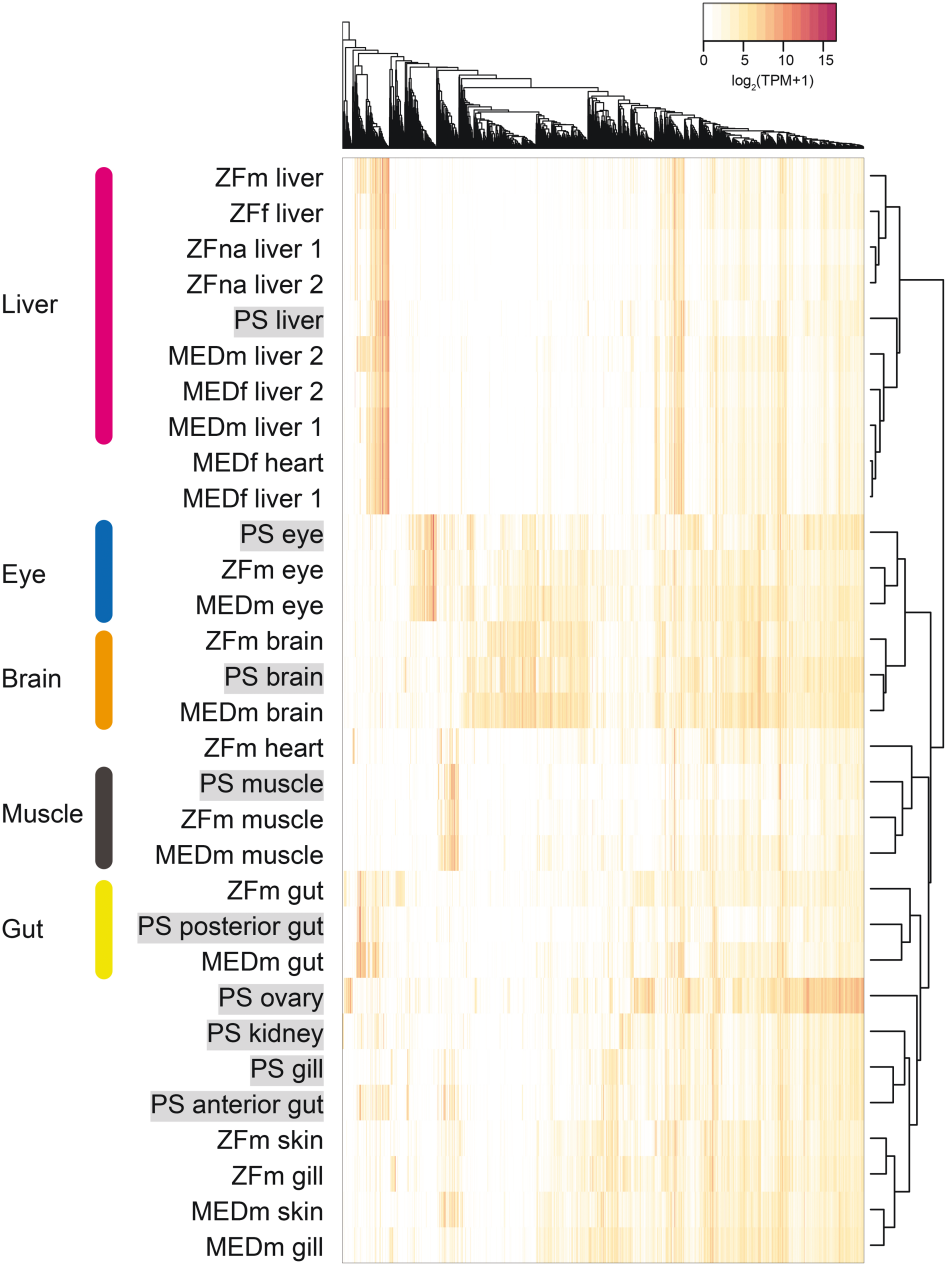
**Clustering of the transcriptome samples.** The heatmap was constructed using the expression data of 4,258 single-copy orthologs, which were expressed in a tissue-specific manner in Pacific saury (PS), zebrafish (ZF), or medaka (MED). For each of ZF and MED tissue samples, the sex information was added: ‘m’ for male, ‘f’ for female, and ‘na’ for ‘not analysed’.

We focussed on tissue-specific genes among single-copy orthologs. Similar to Pacific saury, we predicted the tissue-specific genes among the eight tissues of zebrafish and medaka. These genes were checked against the single-copy ortholog list, and 4,258 of the 8,963 single-copy orthologs were identified as tissue-specific expressed genes in at least one species among Pacific saury, zebrafish, and medaka. Using these genes, we conducted hierarchical clustering of the tissue samples. Based on the heatmap, the expression patterns in the liver, eye, brain, muscle, and gut (posterior gut for Pacific saury) were similar across species (**Fig. 6**). This result suggests that many of the single-copy genes expressed specifically in these tissues may be common among Pacific saury and the model fish species; therefore, these genes may be involved in common functions in each tissue. However, the gill samples were not clustered. As this tissue is directly in contact with water for respiration, the difference in expression profiles might reflect the habitats or sampling environments of these species, namely marine or natural environments for Pacific saury, and laboratory culture for zebrafish and medaka. Accordingly, the skin profiles of zebrafish and medaka were similar to those of the gill, which is also in contact with water.

## 4. Conclusions

In this study, we sequenced the genome and transcriptome of Pacific saury. Based on genome sequencing, we obtained 1.09 Gb of contig sequences by *de novo* assembly, which might have covered most of the genomic sequences of this organism. Most of the ray-finned fish reference genes were captured in the BUSCO assessment, suggesting a high-quality genome assembly. The genome of Pacific saury is relatively rich in repetitive elements, in particular, interspersed repeats, compared to that of medaka in the sister clade, Belonidae, accounting for approximately 60% of the difference in genome size. Using gene prediction, we identified the genes involved in sex steroid biosynthesis, marking the first step in molecular studies on the maturation and reproduction of Pacific saury, a subject that has garnered little interest until now. For transcriptome sequencing, the gene expression levels in nine representative tissues were measured and the genes highly expressed in each tissue were predicted. These genes may be used as markers to characterize the tissues of Pacific saury. In the GO enrichment analysis, tissue-specific genes were associated with the functions of each tissue. Compared with the model fish species, the expression profiles of orthologs were similar among the tissues across the lineage, reflecting common functions in each tissue. Thus, we concluded that the gene expression profiles of Pacific saury tissues are well represented by the current transcriptome data. The results of this study may provide a useful resource for the tissue expression panels of Pacific saury, which will be applicable to further biological and physiological studies on this fish. This research resource will open the door to a comprehensive understanding of transcriptomic responses in Pacific saury to marine environmental changes.

## Supplementary Material

dsae010_suppl_Supplementary_Figure

dsae010_suppl_Supplementary_Table

## Data Availability

The genome and RNA-Seq reads were deposited in the DDBJ database under the BioProject accession number PRJDB16621. The assembled genome and predicted gene sequences of Pacific saury have been deposited in Figshare (DOI: 10.6084/m9.figshare.25060109).
