# Supplementary material for: Draft genome sequence and tissue expression panel of Pacific saury (Cololabis saira)
Source: DNA Res. 2024 Apr 3;31(3):dsae010. doi: 10.1093/dnares/dsae010 (PMC11077904; doi:10.1093/dnares/dsae010)
Supplement: dsae010_suppl_Supplementary_Figure [file dsae010_suppl_supplementary_figure.ppt]

## Slide 1
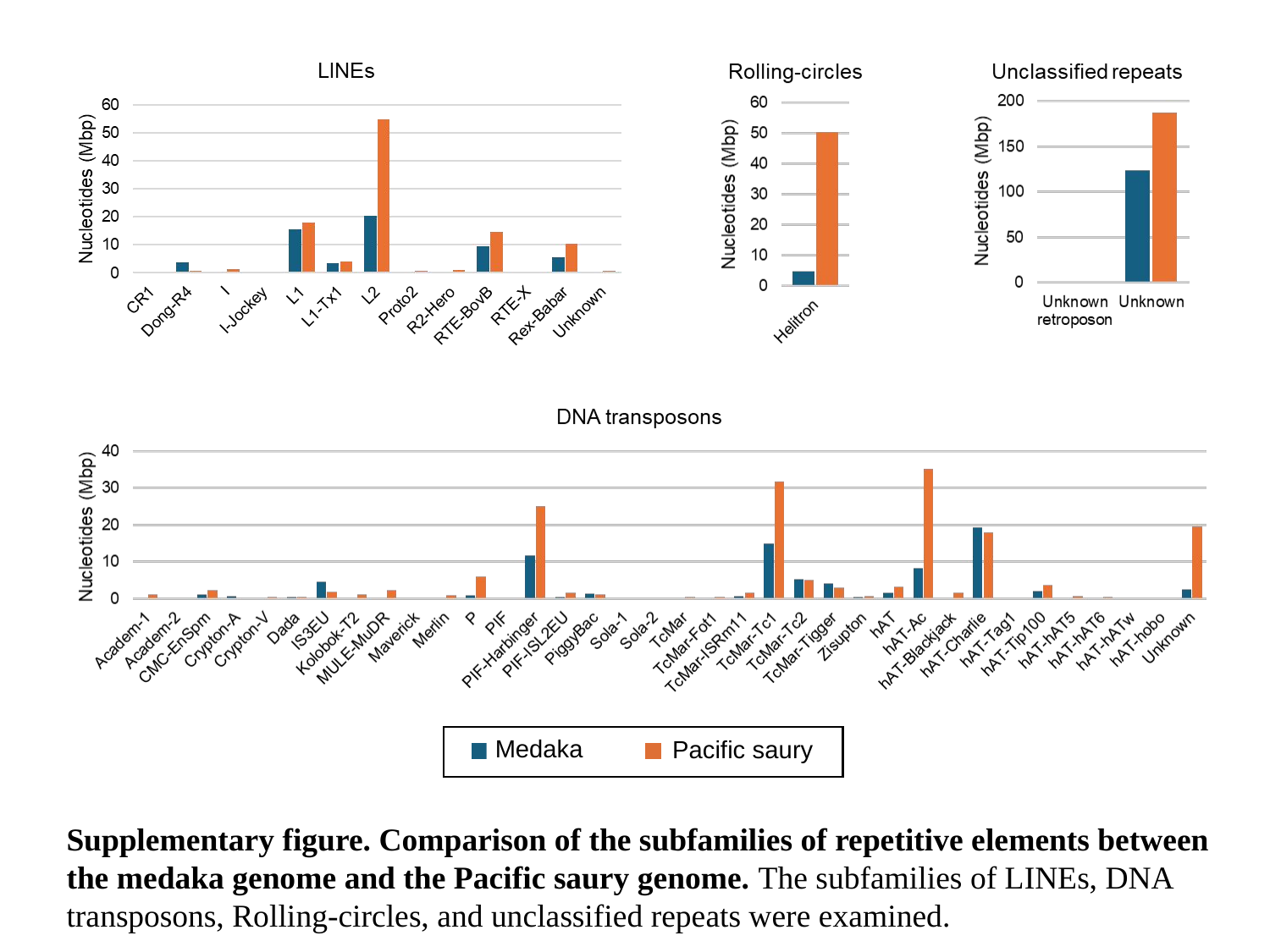

Medaka
Pacific saury
Supplementary figure. Comparison of the subfamilies of repetitive elements between the medaka genome and the Pacific saury genome. The subfamilies of LINEs, DNA transposons, Rolling-circles, and unclassified repeats were examined.
